# Transcriptomic Profile of Directed Differentiation of iPSCs into Hepatocyte-like Cells

**DOI:** 10.3390/ijms27020633

**Published:** 2026-01-08

**Authors:** Irina Panchuk, Valeriia Kovalskaia, Konstantin Kochergin-Nikitsky, Valentina Yakushina, Natalia Balinova, Oxana Ryzhkova, Alexander Lavrov, Svetlana Smirnikhina

**Affiliations:** Research Centre for Medical Genetics, Moskvorechye Str., 1, Moscow 115478, Russiasmirnikhinas@gmail.com (S.S.)

**Keywords:** differentiation, RNA-seq, 3D liver organoids, iPSCs

## Abstract

The liver is the central organ in metabolism; however, modeling hepatic diseases remains limited by current experimental models. Animal models frequently fail to predict human liver physiology, while primary hepatocytes rapidly dedifferentiate in culture. We performed comprehensive transcriptomic profiling of induced pluripotent stem cells (iPSCs) differentiation into hepatocyte-like cells (HLCs) under two-dimensional (2D) and three-dimensional (3D) culture conditions. RNA sequencing analysis revealed the sequential activation of lineage-specific markers across major developmental stages: definitive endoderm (*FOXA2*, *SOX17*, *CXCR4*, *CER1*, *GATA4*), posterior foregut (*PROX1*, *GATA6*), and hepatoblasts (*HNF4A*, *AFP*). Comparative analysis demonstrated a markedly enhanced hepatic gene expression of 3D organoids, as demonstrated by a 33-fold increase in *HNF4A* expression and elevated levels of mature hepatocyte markers, including *ALB*, *SERPINA1*, and *UGT2B15*. However, the 3D cultures retained fetal characteristics (290-fold higher *AFP* expression) and exhibited significantly impaired metabolic function, with *CYP3A4* expression levels reduced by 2000-fold compared to the adult human liver. This partial maturation was further supported by a moderate correlation with adult liver tissue (ρ = 0.57). We demonstrated high reproducibility across five biologically distinct iPSCs lines, including those derived from patients with rare monogenic disorders. The establishment of quantitative benchmarks provides a crucial tool for standardizing in vitro liver models. Furthermore, we delineate the specific limitations of the current model, highlighting the need for further protocol optimization to enhance metabolic maturation and P450 enzyme activity. Functional validation of metabolic activity (CYP enzyme assays, albumin secretion) was not performed; therefore, conclusions regarding hepatocyte functionality are based on transcriptomic evidence.

## 1. Introduction

The liver is an endoderm-derived organ that performs essential endocrine and exocrine functions critical for systemic homeostasis. Liver dysfunction, resulting from a spectrum of diseases including non-alcoholic fatty liver disease (NAFLD), viral hepatitis, cirrhosis, and hepatocellular carcinoma, represents a major global public health challenge. This is due to its high prevalence, therapeutic complexity, and substantial associated healthcare costs [1]. Current models for studying liver disease and preclinical testing models have fundamental limitations. Although widely used, animal models cannot fully recapitulate human liver biology due to significant interspecies differences in metabolism, immune response, and regenerative capacity. Primary human hepatocytes, considered the in vitro gold standard, have two major limitations: rapid functional decline in culture and loss of polarization, and limited availability due to the scarcity of donor organs [2].

**Figure 1:**
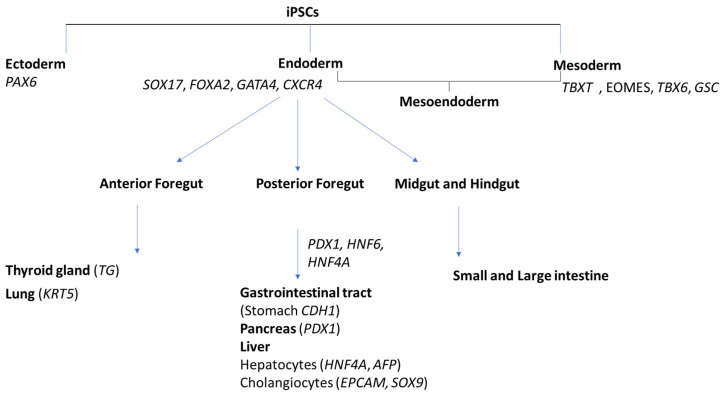
Schematic representation of directed differentiation of induced pluripotent stem cells (iPSCs) into derivatives of the three germ layers and their subsequent tissue specification. Marker genes specific to cell or tissue types are indicated in parentheses and grouped by their germ layer origin.

The development of advanced in vitro liver models has become a major focus in biomedical research. Promising next-generation approaches designed to overcome the limitations of traditional models include 3D liver organoids, microfluidic systems (organ-on-a-chip), and hepatocytes derived from induced pluripotent stem cells (iPSCs). Progress in these models aligns with the global trend of integrating New Approach Methodologies (NAMs), which are supported by regulatory agencies such as the US Food and Drug Administration (FDA) and the European Medicines Agency (EMA). The integration of these advanced models into biomedical research and drug development creates new opportunities to accelerate therapeutic discovery and advance personalized treatment strategies for liver diseases. Furthermore, this approach reduces the ethical and methodological challenges inherent in animal models [3]. The iPSCs platform serves as a powerful tool for preclinical drug screening and development of personalized therapies, providing critical insights into molecular disease mechanisms. iPSC-derived liver organoids demonstrate a high viability and low immunogenic rejection risk, establishing them as a promising tool for regenerative medicine applications [4]. The derivation of hepatocytes from iPSCs recapitulates key stages of embryonic liver development: definitive endoderm (DE), posterior foregut (PF), hepatoblast (LB), and hepatocyte maturation (2D and 3D HLCs). Successful differentiation requires not only the activation of lineage-specific genetic programs but also suppression of alternative developmental fates. The absence of non-hepatic markers confirms precise differentiation control and yields a phenotypically homogeneous culture of hepatocyte-like cells (Figure 1).

The choice of cultivation techniques critically influences hepatocyte functionality and characteristics. Three-dimensional (3D) organoids demonstrate more physiological relevance, morphology, genetic, and cellular similarity to human liver tissue that are superior to conventional two-dimensional (2D) cell models.

Consequently, the 3D approach is more promising for modeling hepatobiliary morphogenesis (development of the liver and gallbladder) and metabolic zonation, as it provides enhanced hepatic gene expression compared to 2D systems. However, a key limitation remains iPSC-derived hepatocytes often retain a fetal phenotype and cannot replicate the full functional spectrum of native liver tissue. This is primarily due to insufficient expression of CYP450 genes, which significantly impairs their xenobiotic metabolism capability [1].

Previously, we developed an optimized protocol for differentiating iPSCs into liver progenitor cells and 3D organoid formation [5]. This study provides a comprehensive characterization of gene expression changes across each differentiation stage. To thoroughly evaluate the differentiation protocol, we performed whole-transcriptome RNA sequencing (RNA-seq) across all stages in both 2D and 3D cultures. A comparative analysis was performed, profiling gene expression between key differentiation stages and with reference human liver RNA sequencing data. The primary objective was to define these transcriptomic benchmarks rather than to assess metabolic functionality, which will be addressed in future studies. Our findings underscore the critical importance of stage-specific profiling for optimizing differentiation protocols and advancing regenerative medicine applications.

## 2. Results

### 2.1. Samples Characterization

**Figure 2:**
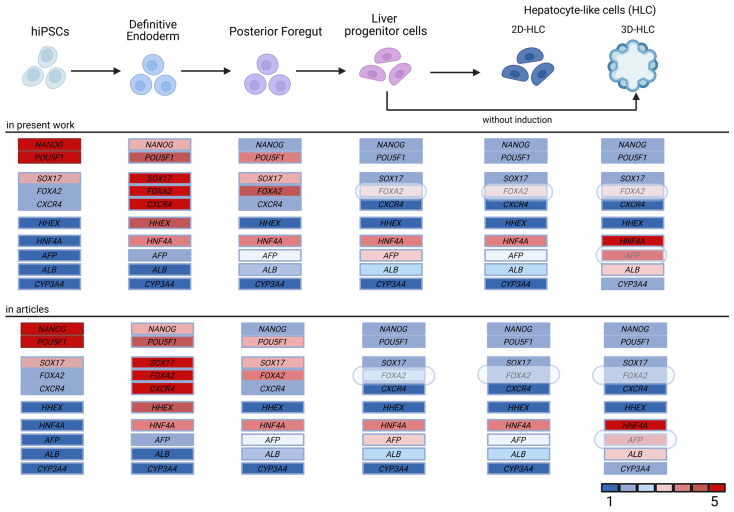
Schematic of the iPSC-to-hepatocyte differentiation protocol and a comparative analysis of key gene expression profiles between our data and published data from other research groups. Differentially expressed genes are highlighted. The color scale indicates relative expression levels, normalized from low (1) to high (5).

**Figure 3:**
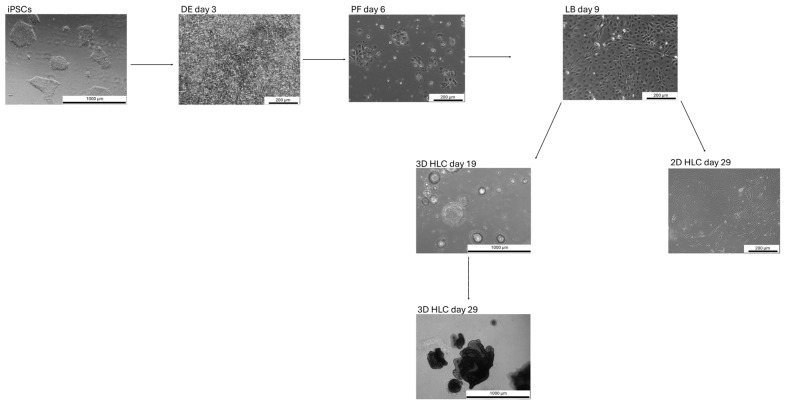
Phase-contrast microscopy images of the differentiation of hiPSCs into hepatocyte-like cells. Scale bars: 200 µm and 1000 µm. iPSCs—induced pluripotent stem cells; DE—definitive endoderm; PF—posterior foregut; LB—liver buds/hepatoblasts; HLC—hepatocyte-like cells, D—day.

**Figure 4:**
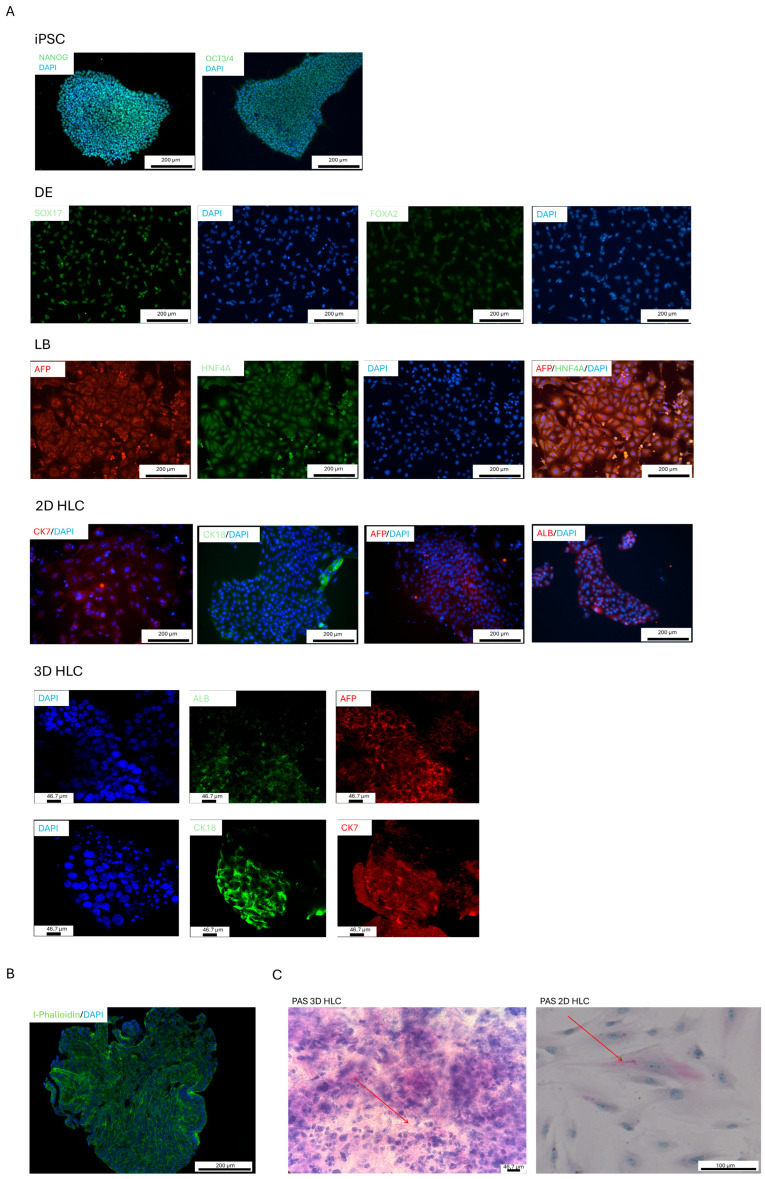
Stage-specific characterization of iPSCs differentiation into hepatocyte-like cells in 3D culture. Immunofluorescence analysis (**A**)—Immunofluorescence analysis of key stages-specific markers. Scale bars 200 µm, 46.2 µm. (**B**)—Visualization of tight junction formation in 3D organoids by Anti-Flotillin-1 immunofluorescence. Scale bar: 200 µm, (**C**)—Periodic acid–Schiff (PAS) staining for glycogen in 2D and 3D hepatocyte-like cells (HLCs). Purple coloration indicates glycogen accumulation. Red arrows highlight representative areas of positive PAS staining in both culture formats, demonstrating the functional glycogen storage capacity of the generated HLCs. Scale bars: 100 µm, 46.7 µm. iPSCs—induced pluripotent stem cells; DE—definitive endoderm; PF—posterior foregut; LB—liver buds/hepatoblasts; HLC—hepatocyte-like cells.

We have previously established an optimized protocol for the directed differentiation of iPSCs into liver progenitor cells, followed by their differentiation into 3D liver organoids (Figure 2 and Figure 3) [5]. This protocol offers several key advantages, including a reduced total differentiation time by 2–3 days and high efficiency, achieved through use of a defined combination of small molecules and growth factors. A simplified differentiation medium, containing only four components for the definitive endoderm (DE), posterior foregut (PF), and hepatoblast (LB) stages, reduces costs and enhances reproducibility. Immunocytochemistry confirmed the high purity of the resulting populations, with 95% of definitive endoderm (DE) cells expressing SOX17 and FOXA2, and 90% of liver progenitor cells expressing HNF4A (Figure 4A). Furthermore, the successful formation of tight junctions in 3D cultures (Figure 4B), visualized using an anti-flotillin-1 antibody, confirmed 3D cell organization (Figure 4C).

The study utilized iPSC lines generated at the Research Centre for Medical Genetics (Russia, Moscow) from two healthy donors and three patients with rare genetic disorders: glycogen storage disease type 1a (OMIM #232200), mucopolysaccharidosis type IVB (OMIM #253010), and type VI (OMIM #253200). Transcriptomic analysis at each differentiation stage enabled us to identify key differentiation points, determination of marker genes for quality control, and monitor dynamic changes in gene expression profiles. The resulting model demonstrated high reproducibility across different cell lines and consistently recapitulated the expected transcriptomic expression patterns at each stage of differentiation (Figure 2 and Figure 3).

**Figure 5:**
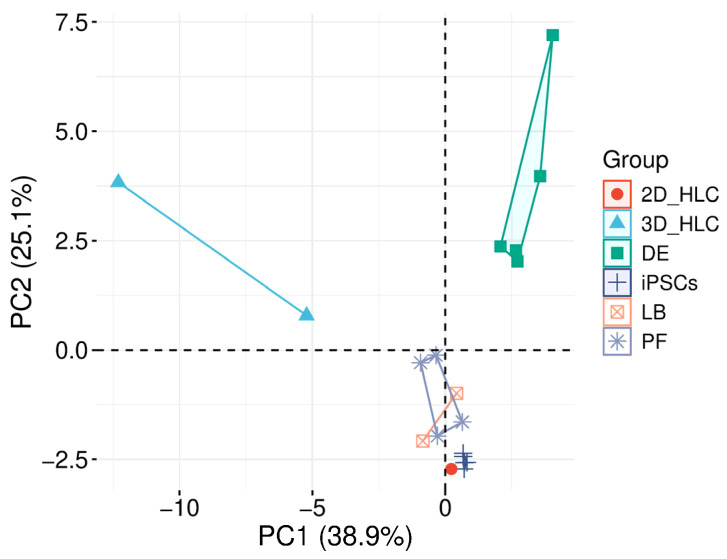
Principal component analysis (PCA) of transcriptomic profiles during iPSCs differentiation into HLCs. The plot demonstrates distinct clustering of the six experimental groups along the first two principal components, which collectively explain 64% of the total transcriptional variance (PC1: 38.9%, PC2: 25.1%). Three-dimensional hepatocyte-like cells (HLCs) and definitive endoderm (DE) clusters show clear separation along PC2, with 3D HLCs being the most distant group, indicating a profound effect of 3D culture on gene expression patterns. iPSCs, posterior foregut (PF), hepatoblast (LB), and 2D HLCs form an overlapping cluster, while DE maintains an intermediate position between early and late differentiation stages.

Transcriptomic profiling by principal component analysis (PCA) identified the transition to three-dimensional (3D) culture as the key factor altering the gene expression profile of hepatocyte-like cells (HLCs). PC1 (38.9% of the variance) separated samples by differentiation stage, while PC2 (25.1% of the variance) revealed a distinct cluster for 3D HLCs, demonstrating transcriptomic difference from both iPSCs and 2D HLCs. These findings conclusively show that 3D HLCs possess a cellular composition significantly distinct from that of 2D cultures (Figure 5).

### 2.2. Expression of Pluripotency Markers

**Figure 6:**
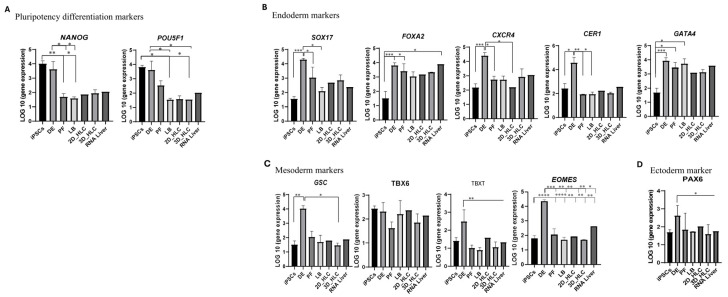
Expression dynamics of lineage-specific markers during iPSCs differentiation into HLCs. Gene expression levels were assessed for pluripotency genes (**A**), endoderm markers (**B**), mesoderm markers (**C**), and ectoderm markers (**D**) Significance levels: *p* < 0.05 (*), *p* < 0.005 (**), *p* < 0.0005 (***), *p* < 0.00005 (****). Abbreviations: iPSCs—induced pluripotent stem cells; DE—definitive endoderm; PF—posterior foregut; LB—liver buds/hepatoblasts; HLC—hepatocyte-like cells; RNA Liver—human liver total RNA.

At the iPSC stage, high expression of the pluripotency markers (*POU5F1*, *NANOG*) confirmed population purity (Figure 4A). As differentiation progressed into definitive endoderm (DE), the expression of these markers declined. This downregulation became statistically significant (*p* < 0.05) at all subsequent stages (PF, LB, 2D-HLC, 3D-HLC), confirming the loss of pluripotency (Figure 4A and Figure 6A).

### 2.3. Expression of Definitive Endoderm Markers

Definitive endoderm (DE) was induced using a combination of Activin A, CHIR99021, and FGFβ. Cells at this stage exhibited characteristic expression of the DE markers *SOX17*, *FOXA2*, *CXCR4*, *CER1*, and *GATA4* (Figure 6B–D). *FOXA2* expression levels remained consistently high throughout all subsequent differentiation stages (Figure 2 and Figure 4A,B). Analysis of other germ layer markers revealed the absence of significant expression of the ectodermal marker *PAX6* at any stage (Figure 6D). Specifically, mesodermal markers (*TBXT*, *EOMES*, *GSC*), showed a brief upregulation at the DE stage, followed by a sharp decline thereafter. The mesodermal marker *TBX6* showed minimal expression throughout the entire differentiation process (Figure 6C).

Transcriptomic analysis revealed a significant upregulation of definitive endoderm markers, including *FOXA2* (232–290-fold increase, *p* < 0.05) and *SOX17* (232–798-fold increase, *p* < 0.05) compared to iPSCs stage [5]. Spearman’s correlation analysis demonstrated positive associations between iPSC and DE stages (ρ = 0.27, *p* < 0.05) and between DE and PF stages (ρ = 0.24, *p* < 0.05). These results confirm the efficient induction of definitive endoderm and posterior foregut cell fate, demonstrating linear consistency across these differentiation stages.

Gene expression analysis identified key parameters for the efficient differentiation of iPSCs into definitive endoderm (DE). Successful differentiation was characterized by significant upregulation of key DE markers (*FOXA, SOX17*, *CXCR4*, *CER1*, *GATA4*), reaching levels approximately 200-fold higher than the iPSC baseline. Our data indicate that balanced suppression of alternative germ layers is critical. Under this protocol, optimal differentiation was defined by specific expression ratios: DE-to-ectoderm genes between 30 and 150 and DE-to-mesoderm genes between 1.7 and 4.4. These findings suggest that maintaining these ratios effectively suppresses alternative differentiation pathways and promotes selective commitment to the endodermal lineage.

### 2.4. Expression of Liver Progenitor Cell Markers

**Figure 7:**
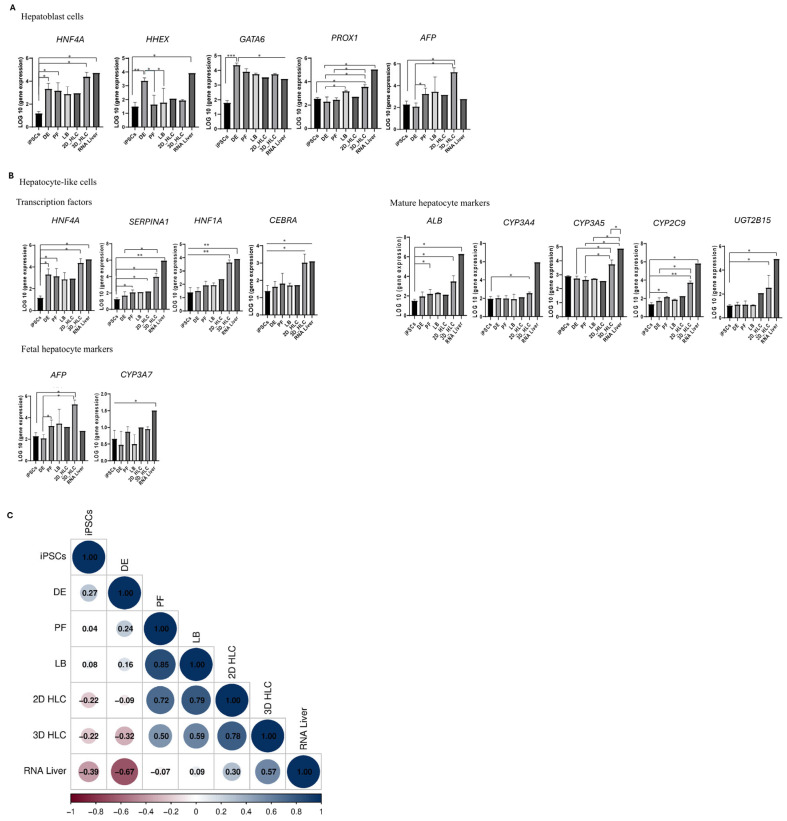
Expression dynamics of lineage-specific markers during iPSCs differentiation into HLCs. Gene expression dynamics of (**A**) hepatoblast markers and (**B**) mature hepatocyte markers during the directed differentiation of iPSCs into hepatocyte-like cells (HLCs). Significance levels: *p* < 0.05 (*), *p* < 0.005 (**), *p* < 0.0005 (***). (**C**) Stage-to-stage correlation analysis, with color scale representing Spearman’s coefficients (red: −1, strong negative correlation; blue: +1, strong positive correlation). Abbreviations: iPSCs—induced pluripotent stem cells; DE—definitive endoderm; PF—posterior foregut; LB—liver buds/hepatoblasts; HLC—hepatocyte-like cells; RNA Liver—human liver total RNA.

Throughout differentiation, we observed the sequential upregulation of key hepatocyte markers *HNF4A*, *AFP*, *PROX1*, and *GATA6* (Figure 7A). The *HNF4A* expression level directly correlated with hepatobiliary maturation. The directed differentiation process recapitulates embryogenesis; therefore, we compared the expression profiles of posterior foregut (PF) and hepatoblast cells (LB) against markers of alternative endodermal lineages. This analysis confirmed predominant commitment to a hepatic fate (Supplementary Figure S1).

Transcriptomic analysis revealed significant upregulation of key liver progenitor cell markers *HNF4A* (61-fold increase, *p* < 0.05) and *AFP* (74-fold increase, *p* < 0.05) compared to the iPSC stage. These transcriptomic findings were confirmed by immunocytochemical analysis, which demonstrated high differentiation efficiency in our previous work. Specifically, 90% of the cell population expressed HNF4A, and 50% of the liver progenitor cells co-expressed HNF4A and AFP (Figure 4B) [5].

Specific gene expression parameters define the successful differentiation of iPSCs into liver progenitor cells. Activation of the hepatic differentiation program requires upregulation of key liver progenitor markers (*AFP*, *HNF4A*, *GATA6*). In our experiments, expression of these markers increased by at least 10-fold compared to the iPSC baseline. The specificity of differentiation was confirmed by a 10- to 15-fold higher expression of these hepatic markers over genes representative of other endodermal lineages. This expression profile promotes commitment to the hepatic lineage while suppressing alternative cell fates. The high correlation between PF and LB stages (ρ = 0.85, *p* < 0.05) confirms a linear differentiation progression and a preferential commitment to the hepatoblast lineage (Figure 7C).

### 2.5. Expression Markers in Hepatocyte-like Cells

In 3D hepatic culture, *HNF4A* expression was 33-fold higher than in 2D HLCs. Similarly, expression of *AFP* and *ALB* was significantly elevated in 3D cultures, showing 290-fold and 5–7-fold increases, respectively. However, both 2D and 3D HLCs exhibited elevated *AFP* levels but reduced *CYP3A4* and *ALB* expression compared to human liver total RNA (Figure 2 and Figure 4A). Notably, *CYP2C9* and *CYP3A5* were expressed at higher levels than *CYP3A4* in both culture systems. The mature hepatocyte markers *SERPINA1* and *UGT2B15* showed increased expression only in 3D HLCs during differentiation, and this trend was not observed in 2D HLCs, where they remain lower than in human liver total RNA (Figure 7C).

In 3D cultures, *HNF4A* expression reached 80% of the level in human liver total RNA. The expression of other key markers differed substantially from the native tissue *AFP* (290-fold higher than liver RNA) and *CYP3A7* (1.7-fold higher than liver RNA), while mature markers were significantly lower expression levels of *ALB* (350–400-fold), *SERPINA1* (13–20-fold), *CYP3A4* (2000-fold), *CYP2C9* (120-fold), *CYP3A5* (10-fold), and *UGT2B15* (120-fold) compared to human liver total RNA (Figure 5).

Spearman’s correlation analysis revealed a strong positive correlation between hepatoblasts (LB) and 2D HLCs (ρ = 0.79, *p* < 0.05), confirming efficient differentiation of progenitor cells into 2D hepatocyte-like cells (Figure 7C). However, this correlation decreased to a moderate level between LB and 3D HLCs (ρ = 0.59, *p* < 0.05), indicating that the 3D architecture promotes a distinct transcriptional profile driven by enhanced cell–cell interactions and unique microenvironmental signaling. Also, immunocytochemistry (Figure 4A) further supported these findings, showing a low expression of ALB, a comparable expression of CK18, CK7, and AFP and reduced Periodic Acid–Schiff (PAS) staining result in 2D HLC culture (Figure 4A,C). Our transcriptomic analysis demonstrates that 3D HLCs exhibit gene expression patterns more closely resembling adult liver tissue compared to 2D counterparts, although both culture systems retain a predominantly fetal hepatic signature.

### 2.6. Bioinformatic Analysis of 2D HLC and 3D HLC Group Compared to RNA Liver

Transcriptomic profiling identified dominant molecular programs during iPSCs differentiation into 2D and 3D hepatocyte-like cells (HLCs), characterized by upregulated expression of genes related to extracellular matrix organization (e.g., *COL1A1*, *COL1A2*, *COL4A1*, *COL4A2*, *COL5A1*, *COL6A1*, *FN1*, *LAMA5*, *VCAN*, *FBN*), molecular regulators of cytoskeleton dynamics (e.g., *FLNA*, *MYH9*, *TPM1*, *AHNAK*), and intercellular signaling (e.g., *PTPRZ1*, *NOTCH3*, *LRP*1). This expression signature—particularly the enrichment of proliferation markers (*MKI67*, *CENPF*)—reflects an active tissue remodeling state consistent with differentiation process.

**Figure 8:**
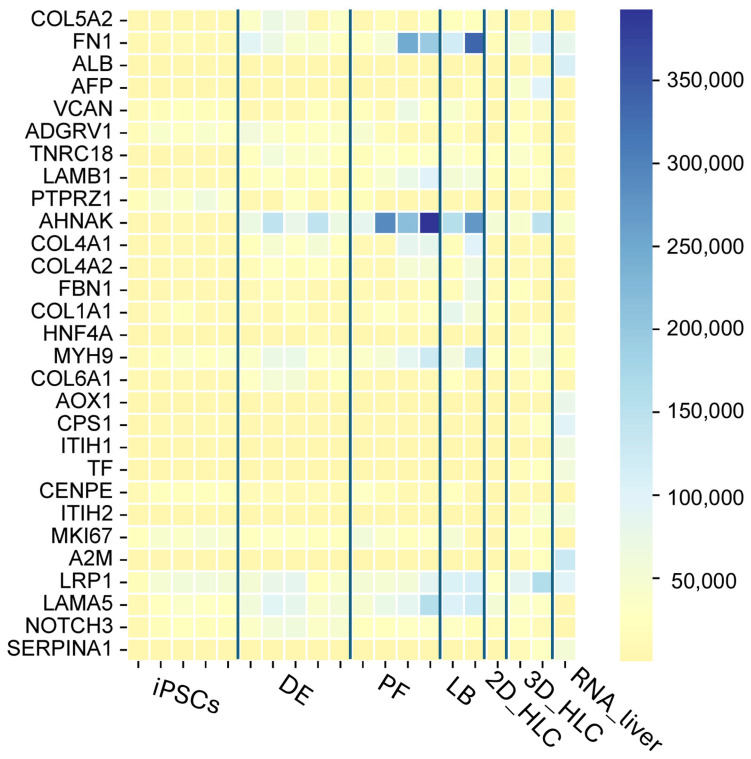
Heatmap of the most variable genes during hepatic differentiation. Expression patterns of genes demonstrate the highest variance across all samples. Rows represent individual genes and columns represent experimental samples. The color key indicates Z-score normalized expression levels, blue colorindicating high expression, while yellow denotes low expression. The analysis identified genes that were most dynamically altered in expression compared to the control (total liver RNA).

The human liver transcriptome exhibited a characteristic functional profile, defined by the expression of genes involved in plasma protein synthesis (*ALB*, *TF*, *A2M*, *ITIH1*, *ITIH2*) and metabolic enzymes (*CPS1*, *AOX1*). Detection of key hepatic markers (*ALB*, *CPS1*) in organoids confirmed successful hepatocytic differentiation, while persistent expression of extracellular matrix genes (*FN1*, *COL1*) suggested retention of a fetal-like phenotype in the cultured cells (Figure 8).

**Figure 9:**
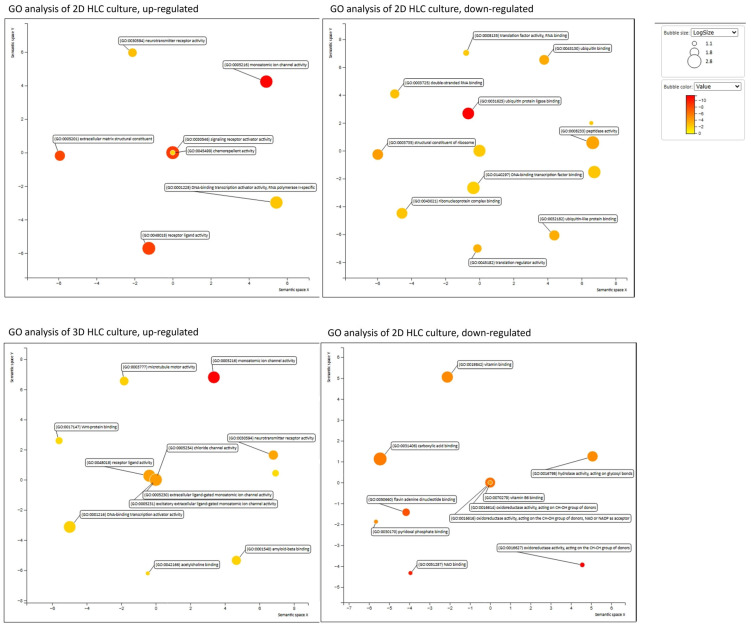
Functional characterization of hepatocyte-like cells through GO enrichment analysis. Scatterplots generated by REVIGO display non-redundant Gene Ontology biological processes for 2D HLCs and 3D HLCs compared to human liver RNA. Redundancy reduction was applied using the “Small (0.5)” setting. Circle color represents the statistical significance (−log_10_ adjusted *p*-value) of the enriched GO terms, while circle size corresponds to the number of genes associated with each term.

Differential expression analysis was performed using DESeq2 by comparing both 2D HLCs and 3D HLCs to human liver RNA. To reveal functional patterns among the identified differentially expressed genes (DEGs), we conducted Gene Ontology (GO) enrichment analysis focusing on the “Molecular Function” category. The results were visualized using SRplot, a freely accessible web server, and REVIGO, which implements a clustering algorithm to reduce semantic redundancy based on similarity measures [6]. The analysis was performed separately for up- and downregulated genes in both comparison groups (2D HLCs vs. human liver RNA and 3D HLCs vs. human liver RNA) (Figure 9).

GO analysis of upregulated genes in 3D HLCs revealed significant enrichment of pathways related to oxidoreductase activity (GO:0016627), correlating with enhanced hepatic gene expression in the 3D culture. In 3D HLCs, terms for ion channel activity (GO:0005216) and, most notably, Wnt-protein binding (GO:0017147) were enriched. Conversely, metabolic pathways governed by ligand-activated nuclear receptors, including PPARα, FXR (GO:0003707), were suppressed. The elevated expression of *AFP* combined with low expression of *ALB* and *CYP3A4* in 3D HLCs suggests that hyperactivation of the Wnt/β-catenin pathway may be a key mechanism maintaining the fetal phenotype in 3D HLCs culture.

GO analysis of 2D HLCs revealed significant negative enrichment in ubiquitin-related pathways, including ubiquitin protein ligase binding (GO:0031625), ubiquitin-like protein ligase binding (GO:0044389), ubiquitin binding (GO:0043130), and ubiquitin-protein transferase activity (GO:0004842). The downregulation of these genes suggests impaired regulation of key cellular processes, such as cell cycle control and apoptosis. Concurrently, we observed enhanced activity in pathways related to extracellular matrix organization conferring tensile strength (GO:0030020) and DNA-binding transcription activator activity (GO:0001216, GO:0001228). This expression signature may indicate the onset of dedifferentiation in the 2D HLC culture.

**Figure 10:**
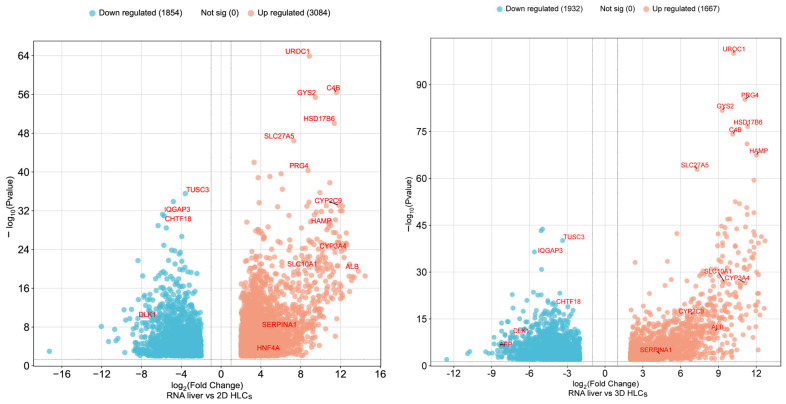
Comparative transcriptomic profiling of 2D-HLCs and 3D-HLCs against human liver RNA. Genes with significant differences in expression levels (|Log_2_FC| > 2, padj < 0.01) are highlighted in color. The names of genes with outlying large differences in expression levels are presented on the diagram. Select genes with the most substantial expression differences are labeled.

Volcano plot analysis was used to visualize differential gene expression in hepatocyte-like cells. The plot displays transcriptomic comparisons of 2D HLCs versus human liver RNA and 3D HLCs versus human liver RNA. Two-dimensional HLCs show an explicit deficiency in key hepatocyte functions, with significant downregulation of genes encoding urea cycle enzymes (*UROC1*), glycogen synthesis (*GYS2*), and major plasma proteins (*ALB*, *ORM1*). Concurrent upregulation of cell-cycle regulators (*LMNB2*, *PKMYT1*) was also observed. While 3D HLCs show partial improvement over 2D HLCs—evidenced by maintained *HNF4A* expression and reduced deficits in key hepatic markers (*ALB*, *CYP3A4*, *CYP2C9*)—they still fail to achieve a mature liver phenotype. Direct comparison of 3D HLCs with human liver RNA confirms persistent expression of the fetal marker *AFP* and suppression of liver-specific metabolic genes (*UROC1*, *HAMP*) (Figure 10).

Differentiation under 3D conditions yields cells with a phenotype more closely resembling primary hepatocytes than 2D HLC cultures, as observed in our experiments. However, the resulting 3D HLCs retain characteristics of a fetal developmental stage and fail to activate the full spectrum of metabolic functions characteristic of mature human hepatocytes. Achieving complete functional maturity requires activation of nuclear receptor-driven programs (PPARα, CAR/PXR, FXR). This study demonstrates that 3D HLCs constitute a valuable yet incomplete model. Our bioinformatic analysis identified the Wnt/β-catenin pathway as the most probable molecular regulator maintaining this immature state.

A strong transcriptional correlation was preserved between 2D and 3D hepatocyte-like cells (ρ = 0.78, *p* < 0.05), confirming their shared developmental trajectory from a common progenitor population. Notably, 3D HLCs demonstrate a moderate correlation with human liver tissue (ρ = 0.57, *p* < 0.05) that substantially exceeded the weak correlation observed for 2D cultures (ρ = 0.30, *p* < 0.05). The lack of significant correlation between hepatoblasts (LB) and human liver tissue (ρ = 0.09) underscores their immature status (Figure 7C). To investigate the molecular basis of the fetal phenotype observed in the cultures, we performed Gene Ontology (GO) analysis, which revealed hyperactivation of the Wnt/β-catenin signaling pathway. These findings indicate that 3D organization promotes a more closely resembling native tissue and superior preservation of native tissue-like microarchitecture compared to 2D cultures.

## 3. Discussion

In this study, we investigated the stepwise directed differentiation of iPSCs into hepatocyte-like cells by analyzing transcriptomic changes across all key developmental stages. We established quantitative gene expression benchmarks for successful lineage commitment. Activation of the endodermal program was defined by an approximately 200-fold upregulation of markers *FOXA2*, *SOX17*, *CXCR4*, *CER1*, and *GATA4*. Differentiation into hepatic progenitors was marked by a 10-fold increase in expression of *HNF4A* and *AFP* compared to earlier stages. Comparative profiling revealed the superior enhanced hepatic differentiation of 3D organoids, which demonstrated markedly elevated expression of key hepatic markers—including *HNF4A* (33-fold), *AFP* (290-fold), and *ALB* (5–7-fold) relative to their 2D HLCs culture.

Our results demonstrate that the differentiation protocol accurately reproduces key stages of physiological hepatogenesis. The established quantitative criteria provide a foundation for standardizing differentiation methods and generating more specific cellular models for liver pathologies, and refining protocols to produce more enhanced differentiation iPSC-derived hepatocytes for drug evaluation.

Our data demonstrates a sequential decrease in the expression of pluripotency markers (*POU5F1*, *NANOG*) during the differentiation iPSCs into HLCs (Figure 6). Expression continued to decline throughout all subsequent stages (PF, LB, 2D/3D-HLC), with the most significant downregulation occurring in 3D culture conditions. While the specific dynamics of this downregulation may be protocol-dependent, our data robustly validate earlier reports in the field [7,8,9].

Definitive endoderm (DE) induction was achieved using a highly efficient protocol combining Activin A and CHIR99021 for the first 24 h, followed by FGFβ supplementation. Activin A, an agonist of the TGF-β/SMAD pathway, activates ALK4/7 receptors, leading to SMAD2/3 phosphorylation and subsequent induction of key DE markers SOX17 and FOXA2. CHIR99021, GSK-3β inhibitor, promotes β-catenin stabilization and WNT pathway activation, thereby enhancing the effects of Activin A [10,11,12]. FGFβ acts through FGFR1 to activate the MAPK cascade, supporting DE cell proliferation and survival while upregulating endodermal markers, including *FOXA2*, *SOX17*, *CXCR4*, *CER1*, and *GATA4* (Figure 4A). These findings are supported by our transcriptomic data and align with published studies [7,12,13]. Unlike other markers, *FOXA2* expression remained consistently high throughout differentiation (Figure 2), an effect attributable to the specific combination of small molecules and growth factors used in our protocol [10]. This persistent *FOXA2* expression may indicate retained progenitor multipotency and arrested maturation. Analysis of other germ layer markers, ectoderm (*PAX6*) and mesoderm (*TBXT*, *EOMES*, *TBX6*, *GSC*) (Figure 6B,C), revealed no ectodermal upregulation after the DE stage. Mesodermal markers showed transient expression peaks at the DE stage, followed by a sharp decline (except *TBX6*, which had minimal initial expression). This pattern recapitulates mesoendoderm formation and subsequent endodermal commitment, which is consistent with the current understanding of endoderm development [7,14].

Our data confirms that the hepatic phenotype is established stepwise through posterior foregut and hepatoblast stages, marked by characteristic changes in marker expression. This process is defined by increased expression of key markers: *HNF4A*, *AFP*, *PROX1*, and *GATA6* (Figure 7A). Among these, *HNF4A* is a master regulator of hepatobiliary commitment. The upregulation of *AFP* is specific to the hepatocyte lineage and is not observed during cholangiocyte specification. To direct differentiation toward a hepatic fate, we used a combination of growth factors: FGF10 and small molecules (SB431542 (an inhibitor of the TGF-β signaling pathway) and retinoic acid. FGF10 binds to the FGFR2b receptor (FGF receptor 2 isoform IIIb), activating the intracellular PI3K-AKT and MAPK pathways. This signaling ensures the development of hepatic, rather than pancreatic, progenitor cells [15]. The small molecule SB431542 inhibits the SMAD2/3 signaling pathway by ALK4/5/7 receptors, thereby preventing commitment to alternative differentiation lineages. Retinoic acid, acting through RAR/RXR nuclear receptors, induces the expression of genes critical for hepatic precursor, including *HHEX*, *HNF1A*, and *HNF4A*. FGF10 and BMP4 play pivotal roles at the hepatoblast (LB) stage. FGF10 promotes proliferation via MAPK pathway activation, while BMP4 activates the SMAD1/5/9 pathway. This synergy interaction upregulates characteristic hepatoblast markers (*HNF4A*, *PROX1*, *AFP*) while suppressing cholangiocyte development, as evidenced by low *SOX9* expression (Supplementary Materials, Figure S1). Thus, the coordinated increase in expression levels of *HNF4A*, *AFP*, and *PROX1* serves as a reliable indicator of successful differentiation into liver progenitor cells. This expression profile is consistent with established differentiation protocols in the literature [9,16,17,18].

To confirm the specificity of hepatic differentiation, we examined the expression of markers for other endodermal derivatives. At the liver progenitor cell stage, no significant upregulation was detected for markers of pancreatic acinar cells, gastrointestinal enterocytes/endocrine cells, lung alveolar/bronchial epithelium, or thyroid follicular cells compared to the human liver total RNA (Supplementary Materials, Figure S1). The analysis revealed increased expression of pancreatic marker *PDX1* (pancreatic β-cells marker) and the cholangiocytes marker *EPCAM*. These data suggest the formation of a heterogeneous cell population with pancreato–biliary–hepatocytic differentiation potential. However, detection of *PDX1* and *EPCAM* does not constitute definitive evidence of mature pancreatic cells or cholangiocytes formation, as both markers are also expressed in anterior–posterior foregut progenitor cells that retain bipotential differentiation capacity. Furthermore, expression levels of these genes do not directly correlate with functional protein production.

The key markers for assessing functional maturity in 3D HLCs include genes encoding specialized hepatocyte functions. The transcription factor HNF4A plays a central role in the differentiation and maintenance of hepatocyte identity by regulating liver-specific gene expression; its loss leads to hepatocytes dedifferentiation with the loss of their specialized functions [19]. Alpha-fetoprotein (AFP) is a fetal marker, while albumin (ALB) is a major functional protein synthesized by mature hepatocytes. Critical markers for assessing expression of metabolic genes include cytochrome P450 genes (*CYP3A4*, *CYP3A5*, *CYP2D9*) and UDP-glucuronosyltransferase (*UGT2B15*), which play essential roles in xenobiotic metabolism. We compared the transcriptomic profiles of 2D and 3D HLCs with human liver total RNA at the final differentiation stage, enabling cross-referenced analysis of their maturation status.

Under 3D conditions, expression of *HNF4A*—a master regulator of hepatocyte differentiation—reached 80% of the level in human liver RNA, consistent with its established critical role in maintaining the mature hepatocyte phenotype [19]. Analysis of albumin (*ALB*) expression proved particularly revealing as a key marker of hepatocyte transcriptomic maturation, *ALB* levels in 3D culture exceeded those in 2D culture by 5–7-fold. This enhancement can be explained by higher expression of its key transcriptional regulators (*HNF4A*, *CEBPA*, *GATA4* and *HNF1A*) under 3D conditions. These findings align with other studies demonstrating the advantages of 3D culture for hepatocyte differentiation [20,21].

The 3D microenvironment supports both the maturation and long-term functional maintenance of HLCs, preventing dedifferentiation and preserving their polarized architecture. Compared to 2D models, 3D HLC culture demonstrates higher expression levels of hepatocyte-specific genes, a finding confirmed by other research groups [21,22]. This is likely due to 3D models’ ability to recapitulate key features of an in vivo hepatic microenvironment, including cell polarization, intercellular contact formation, and enhanced autocrine and paracrine signaling that coordinate and promote differentiation [9,19,20].

We evaluated the expression of key hepatocyte markers *HNF4A*, *AFP*, *ALB*, *SERPINA1*, *CYP3A4*, *CYP2D9*, *CYP3A5*, *CYP3A7*, and *UGT2B15* in 3D HLCs (Figure 4). The transcriptomic profile revealed a mixed phenotype: elevated *HNF4A* and *AFP* alongside low expression of the mature markers *ALB* and *CYP3A4* compared to human liver total RNA. This profile suggested limited predominantly fetal transcriptomic signature and low synthetic activity in the 3D organoids (Figure 2) [10,19]. Fetal phenotype was further indicated by elevated expression of *CYP2C9* and *CYP3A5* relative to *CYP3A4*, a pattern characteristic of fetal liver. Our findings regarding *ALB* and *CYP3A4* expression levels are consistent with reports by Kim et al. [23] and Wang et al. [24]. The progressive upregulation metabolic genes *SERPINA1* and *UGT2B15* during differentiation confirms that the organoids achieved elevated expression of some hepatocyte-associated genes (Figure 7) [10,18,19,25].

The observed transcriptional profile aligns with a fetal hepatic signature, consistent with the experimental design. The primary objective was to establish a robust iPSC-to-HLC differentiation protocol rather than to induce xenobiotic metabolic activity; consequently, no targeted cytochrome P450 (CYP) gene induction was performed. Baseline CYP expression levels corresponded with those reported for non-induced HLCs [26,27,28], further validating the model’s consistency with established protocols.

Current strategies to overcome this limitation focus on the directed differentiation of the cellular program through three complementary approaches: biochemical modulation of key signaling pathways, recreation of the hepatic microenvironment, and the application of bioengineering platforms.

For evaluating xenobiotic metabolism in HLCs, achieving approximately 20% of *CYP3A4* expression level found in primary human hepatocytes (PHHs) may be sufficient [29,30]. Standard protocols, including the one employed here, often activate mitogenic and proliferative signals (e.g., the canonical Wnt-pathway) to expand progenitor pools. This ultimately acts as a barrier to achieving a fully mature phenotype. Therefore, a critical step is the controlled suppression of these pathways during late differentiation stages. This can be achieved by adding small-molecule inhibitors, such as IWP-2 (which suppress Wnt ligand secretion) or IWR-1 (which stabilizes the β-catenin degradation complex) [31,32]. Furthermore, suppressing elevated Wnt/β-catenin signaling—a hallmark of fetal hepatocytes—can also be advanced using specific molecules like FGF19, BMP7, or dexamethasone [31,32]. This modulation enables the balance from proliferation toward the activation of genes responsible for specialized functions, including *ALB* and *CYP3A4*. The induction of *CYP3A4* can be further enhanced by receptor agonists such as rifampicin [33]. Consequently, a key principle is the removal of mitogenic stimuli alongside the addition of maturation factors, creating optimal conditions for functional maturation [34].

The functional maturity of hepatocytes in vivo is maintained by paracrine signals from non-parenchymal liver cells. Replicating this niche in vitro is an effective method for improving differentiation. Co-culturing HLCs with liver sinusoidal endothelial cells, hepatic stellate cells, or mesenchymal stromal cells provides the necessary contact-mediated and humoral signals that promote polarization via growth factors (VEGF, HGF), phenotype stabilization, and enhanced metabolic activity [35,36]. Studies such as the work by Berger et al., which utilized a micropatterned co-culture system with multiple cell types, demonstrate a significant improvement in HLC function, achieving up to 90% of the *CYP3A4* activity observed in primary human hepatocytes (PHHs) [37]. Transitioning to 3D cultures within defined hydrogels based on recombinant extracellular matrix proteins (e.g., laminin combinations with collagen-III, fibronectin, tenascin C, or hyaluronic acid) promotes improved morphology and function of HLCs [38]. Further progress is associated with using microfluidic “organ-on-a-chip” systems, which provide continuous perfusion, generate physiological shear stress, and enable spatially organized co-culture. These conditions substantially enhance the expression of hepatocyte-associated markers compared to standard methods [39,40,41,42]. Notably, as indicated in the literature, merely extending the culture duration without optimizing these conditions does not lead to substantial maturation [10].

Thus, the challenge of fetal immaturity in HLCs necessitates a comprehensive approach that sequentially addresses the regulation of intracellular signaling, cell–cell interactions, and the physicochemical parameters of the environment. The results presented in this work clearly identify the transcriptomic hallmarks of an immature state, defining targets for further optimization. The most effective strategy for subsequent research appears to be a combination of: (1) temporary inhibition of proliferative pathways (Wnt/β-catenin) during the final differentiation stage, (2) integration of non-parenchymal liver cells into the model to establish parenchymal–stromal interactions, and (3) adaptation of the protocol to conditions of 3D perfusion culture. Such a multi-level approach represents a promising pathway for generating functionally competent HLCs suitable for biomedical applications.

Limitations: Several limitations of this study should be considered. First, this work focused on establishing transcriptomic benchmarks for protocol validation. Consequently, direct functional assessments (e.g., CYP450 enzyme activity, albumin secretion), which are essential for confirming hepatocyte functionality, were not performed and remain a priority for future research. Second, the analysis was limited to a single differentiation endpoint; long-term stability experiments were not conducted. Third, although bioinformatic analysis implicates the Wnt/β-catenin pathway in maintaining the fetal transcriptomic signature, its direct causal role was not experimentally validated. Therefore, while this study provides robust transcriptional criteria for differentiation quality, the predictive utility of this platform for drug metabolism studies requires future validation through functional assays. Addressing these points in subsequent work will be essential to fully assess the model’s potential for pharmacological applications.

## 4. Materials and Methods

### 4.1. hiPSC’s Cell Line

Skin fibroblasts from three patients with rare monogenic disorders (P10L1 (OMIM #253010), P17L16 (OMIM #232200), and P11L3 (OMIM #253200)) and two healthy donors (P16L4 and P12L3) were reprogrammed using the CytoTuneTM-iPS 2.0 Sendai Reprogramming Kit (Thermo Fisher Scientific, Waltham, MA, USA) according to the manufacturer’s specifications. The resulting iPSC lines were maintained on Matrigel-coated (Corning, Corning, NY, USA) plates and cultured in TeSR-E8 medium (STEMCELL Technologies, Vancouver, BC, Canada), with daily medium changes to support their growth and pluripotency. Pluripotency was validated through immunofluorescence analysis using antibodies against SSEA-4 (Thermo Fisher Scientific, USA), NANOG (Thermo Fisher Scientific, USA), and OCT-4 (Abcam, Cambridge, UK). Multilineage differentiation potential was confirmed via directed differentiation, demonstrating expression of ectodermal (beta III tubulin (Abcam, UK)), mesodermal (brachyury (eBioscience, San Diego, CA, USA)) and endodermal (FOXA2 (Abcam, UK)) markers. All cell lines tested negative for mycoplasma contamination [43,44,45].

### 4.2. Directed Differentiation

iPSCs were harvested using Versene solution (PanEco, Moscow, Russia) and seeded onto Matrigel-coated 12-well plates (SPL, Gyeonggi-do, Republic of Korea) at a density of 100,000 per cm^2^. Differentiation was initiated following a sequential protocol. Definitive endoderm (DE) was induced for 24 h in basal medium (RPMI 1640 Medium (STEMCELL Technologies, Canada), supplemented with 1% B-27 without Vitamin A (Thermo Fisher Scientific, USA), 1% GlutaMAX (Thermo Fisher Scientific, USA), 1% sodium pyruvate (PanEco, Russia)), 100 ng/mL Activin A (STEMCELL Technologies, Canada), and 3 uM CHIR99021 (Tocris, Bristol, UK). This was followed by three days of culture with 100 ng/mL Activin A and 10 ng/mL FGFβ (PanEco, Russia). Posterior foregut specification was achieved using basal medium supplemented with 50 ng/mL FGF10 (R&D System, Minneapolis, MN, USA), 10 uM SB431542 (Tocris, UK), and 10 uM retinoic acid (Sigma Aldrich, St. Louis, MO, USA). Hepatocyte liver progenitor cells (hepatoblasts) were then maintained in basal medium containing 50 ng/mL FGF10 and 10 uM BMP4 (R&D System, USA) with daily medium changes. For 3D differentiation, the HepatiCult Organoid kit (STEMCELL Technologies, Canada) was used according to the manufacturer’s instructions. Two-dimensional hepatocyte-like cells (2D-HLCs) were generated using identical conditions without matrix embedding. All cultures were maintained at 37 °C, 5% CO_2_, and >90% humidity.

### 4.3. RNA Extraction and Quality Control

Cells were collected at each stage of the directed differentiation protocol: iPSCs, definitive endoderm (DE), posterior foregut (PF), hepatoblasts (LB), and both 2D and 3D hepatocyte-like cells (HLCs). Samples were harvested from one well of a 12-well plate at 70% confluence (approximately 100,000–120,000 cells per cm^2^). Total RNA was extracted using the QIAamp RNA Blood Mini Kit or QIAzol (Qiagen, Germantown, MD, USA), followed by quantification on a Nano-500 spectrophotometer (Allsheng, Hangzhou, China) and assessed quality through 1% agarose gel electrophoresis and capillary electrophoresis (TapeStation 4200, Agilent, Santa Clara, CA, USA). Only samples with quality thresholds RNA integrity numbers (RIN) > 6, concentrations > 50 ng/µL, 260/280 ratios ~2.0, and 260/230 ratios > 2.0 were processed further. Ribosomal RNA was depleted using the Ribo-off rRNA Depletion Kit (Vazyme, Nanjing, China). Human Liver Total RNA (AM7960, Invitrogen, Carlsbad, CA, USA) was used as positive control for RNA sequencing.

### 4.4. Library Preparation and Sequencing

RNA libraries were prepared with the MGIEasy RNA Library Prep Kit (MGI, Shenzhen, China), with coding sequences enriched using the NEXome Plus Panel v1.0 (Nanodigmbio, Nanjing, China). Library quality was assessed by verifying size distribution (~330 bp) using TapeStation DNA ScreenTape (Agilent, USA), and quantifying concentration with the Qubit 2.0 dsDNA High Sensitivity Assay Kit (Invitrogen, USA). Paired-end sequencing (150 bp) was performed on the DNBSEQ-G400 platform (MGI, China) using the manufacturer’s high-throughput sequencing reagents DNBSEQ-G400 High-throughput Sequencing Set.

### 4.5. Bioinformatics Analysis

Alignment and Quantification: Demultiplexed reads were aligned to the GRCh38 (hg38) reference genome (Ensembl v.84) using HISAT2 (with SNP/transcript-aware indexing) [46,47]. Duplicate reads were marked with Picard MarkDuplicates (v3.0.0), and gene-level counts were generated using featureCounts [46] with the following parameters: ignoreDup (exclude duplicates), p –countReadPairs (paired-end mode), t gene -g gene_name (HGNC symbols).

Quality metrics were assessed with FastQC (v 0.12.0, https://www.bioinformatics.babraham.ac.uk, accessed on 3 November 2025) and aggregated using MultiQC (v1.25.2, https://multiqc.info), the number of reads was 70–200 million per sample. Differential expression analysis was performed using DESeq2 [48,49]. Raw counts were normalized, and low-expression genes (<10 reads in ≥1 sample group) were filtered out. Differentially expressed genes (DEGs) were defined as those with |log_2_ fold change| > 1 and adjusted *p*-value < 0.05. Downstream analysis included Spearman’s correlation, Heatmap, Gene Ontology (GO) enrichment visualization using SRplot (https://bioinformatics.com.cn/en, accessed on 3 November 2025) [50].

### 4.6. Immunocytochemistry

Cells were fixed using 3.7% paraformaldehyde (Carl Roth, Karlsruhe, Germany) for 20 min at room temperature (RT), then permeabilized with 0.25% Triton X-100 (Helicon, Moscow, Russia) and blocked with 1% bovine serum albumin (BSA) (Sigma Aldrich, USA). Subsequently, primary and secondary antibodies were incubated for one hour in the dark and 30 min at room temperature, respectively. Antibody details are provided in Table 1. Nuclei were stained with DAPI (Abcam, UK) for 10 min at room temperature. Tight junction formation in 3D cultures was assessed by using Anti-Flotillin-1 immunofluorescence (abnova, U0281) Imaging was performed with a Lionheart FX Automated Microscope (Agilent BioTek, Santa Clara, CA, USA).

### 4.7. Statistical Analysis

All statistical analyses were performed using GraphPad Prism version 9.1.1. Gene expression data were analyzed using the Kruskal–Wallis test with Benjamini–Hochberg false discovery rate (FDR) correction for multiple comparisons. Statistical significance was defined as *p* < 0.05.

### 4.8. Study Design and Scope

The primary objective of this study was to establish transcriptomic benchmarks for the quality control of iPSC differentiation into HLCs. Therefore, the experimental design focused on RNA sequencing and immunocytochemical characterization. Assessment of metabolic functionality (e.g., CYP enzyme activity, albumin secretion) was beyond the scope of this work and will be addressed in future studies.

## 5. Conclusions

This study confirms that our established cellular model reliably recapitulates key stages of hepatocyte differentiation in vitro. Transcriptomic profiling revealed the sequential activation of stage-specific markers: *SOX17*, *FOXA2*, and *CXCR4* in definitive endoderm stage; *PROX1* and *GATA6* in PF cells; and *HNF4A* and *AFP* in LB cells. We defined quantitative benchmarks for successful differentiation, including 200-fold upregulation of key DE markers *FOXA2*/*SOX17*/*CXCR4* relative to iPSCs, with specific DE-to-ectoderm (30–150) and DE-to-mesoderm (1.7–4.4) expression ratios. For hepatoblast cells, a 10-fold induction of *AFP*/*HNF4A*/*GATA6* with 10- to 15-fold higher expression over other endodermal lineages, which ensured the reproducible generation of target cell population.

The resulting 3D organoids exhibited a fetal-like phenotype, characterized by elevated expression of *AFP*, *CYP3A5*, and *CYP2C9*, but low levels of *CYP3A4* and *ALB* demonstrate a profile consistent with existing in vitro liver models. Although these hepatocyte-like cells retained fetal characteristics, they demonstrated partial transcriptomic maturation, as evidenced by the expression of *ALB* and *UGT2B15*. However, their metabolic capacity remained limited due to insufficient cytochrome P450 expression, which positioned the 3D HLCs at an intermediate stage between fetal and adult liver. Though not fully equivalent to native tissue, this model is suitable for studying liver pathologies and drug screening, provided its limitations are considered. These findings highlight the importance of selecting disease-specific models and the need for continued protocol refinement to achieve full functional maturation.

The established protocol, utilizing a defined combination of small molecules and growth factors, effectively directs the differentiation of iPSCs into hepatic progenitor cells and their subsequent organization into 3D structures. Further improvements could focus on enhancing metabolic function by inducing cytochrome P450 enzyme activity. It should be noted that functional assays (CYP activity, albumin secretion) were not performed in this study; therefore, the utility of these HLCs for drug metabolism studies requires further validation. Thus, this study provides a robust and reproducible platform for fundamental and applied research in the field of liver biology studies.

## Figures and Tables

**Table 1:** List of used antibodies.

Antibodies	Source	Identifier
Rabbit anti-SOX17	Abcam, UK	Cat#ab224637; RRID: AB_2801385
Rabbit anti-HNF4A	CΦ	Cat#A20865; RRID: AB_2728751
Rabbit anti-FOXA2	Abcam, UK	Cat#ab108422; RRID:AB_11157157
Mouse Anti-Alpha-Fetoprotein (AFP)	Abclonal, USA	Cat#A17898; RRID:AB_2861748
Rabbit Anti-Albumin	Abcam, UK	Cat#ab106582;RRID:AB_10888110
Rabbit Anti-Cytokeratin 18	Abcam, UK	Cat#ab133263; RRID:AB_11155892
Mouse Anti-Cytokeratin 7	ELK biotechnology, Buckingham, UK	Cat#EM1054; RRID:AB_
Goat anti-Mouse IgG (H + L), Alexa Fluor 594	Thermo Fisher Scientific, USA	Cat#A-11032; RRID:AB_2534091
Goat anti-Rabbit IgG (H + L), Alexa Fluor 594	Thermo Fisher Scientific, USA	Cat#A-11037; RRID:AB_2534095
Goat anti-Rat IgG (H + L), Alexa Fluor 488	Abcam, UK	Cat#ab150113; RRID:AB_2576208
Anti-Rabbit IgG H&L, Alexa Fluor 488	Abcam, UK	Cat#ab150077; RRID:AB_2630356

## Data Availability

The raw RNA-seq data are available in the NCBI Sequence Read Archive (SRA) under the BioProject accession PRJNA1346313.

## Supplementary Materials

The following supporting information can be downloaded at: https://www.mdpi.com/article/10.3390/ijms27020633/s1.
