# Supplementary material for: Transcriptomic Profile of Directed Differentiation of iPSCs into Hepatocyte-like Cells
Source: Int J Mol Sci. 2026 Jan 8;27(2):633. doi: 10.3390/ijms27020633 (PMC12840978; doi:10.3390/ijms27020633)
Supplement: Supplementary file 1 [file ijms-27-00633-s001.zip › ijms-3965233-supplementary.pdf]

Comparative marker profiling validates the lineage specificity of iPSC-derived hepatic differentiation.

The directed differentiation process accurately models recapitulate key stages of embryonic liver development. To evaluate lineage specificity, we performed comparative transcriptomic profiling against established markers of alternative endodermal lineages (pancreatic, intestinal, pulmonary, and thyroid).

The analysis demonstrated robust hepatic specification with no significant upregulation of markers for pancreatic acinar cells, gastrointestinal tract, pulmonary epithelium, or thyrocytes. Absolute expression levels of these non-hepatic markers remained low, showing minimal expression comparable to baseline hepatic levels (Supplementary Figure 1).

Although, we observed statistically significant stage-specific modulation of pancreatic progenitor marker *PDX1* and cholangiocyte marker *EPCAM* ($p<0.05$), absolute *PDX1* transcript levels remained within baseline thresholds. These results confirm generation of developmentally relevant hepatoblasts with bipotent capacity for both hepatocytic and cholangiocyte commitment.

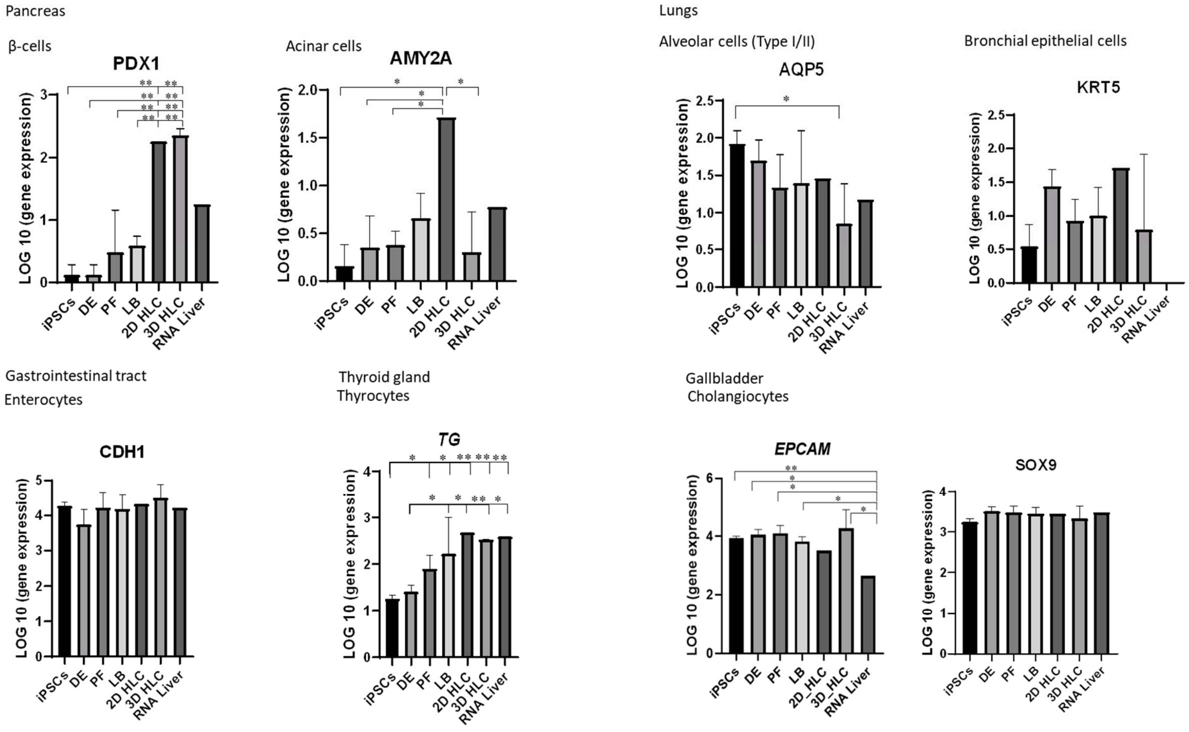

Figure S1. Expression of lineage-defining markers of pancreatic, gastrointestinal, lung, and thyroid cell lineages. Gene expression profiles demonstrate specificity of hepatic differentiation. Significance levels: $p<0.05$ (*), $p<0.005$ (**). Abbreviations: iPSCs — induced pluripotent stem cells; DE — definitive endoderm; PF — posterior foregut; LB — hepatoblasts; HLC—hepatocyte-like cells; RNA Liver — total liver RNA.
